# Pulmonary artery sarcoma resected via median sternotomy with thoracoscopic assistance

**DOI:** 10.1186/s40792-016-0279-6

**Published:** 2017-01-09

**Authors:** Hiromichi Katakura, Yojiro Yutaka, Kenichi Takahashi, Tsuyoshi Shoji, Akira Yamanaka, Mitsuru Kitano

**Affiliations:** Department of Thoracic Surgery and Respiratory Medicine, Department of Cardiovascular Surgery, Otsu Red Cross Hospital, Nagara 1-1-35, Otsu, Shiga 520-8511 Japan

## Abstract

Median sternotomy is frequently selected for the resection of pulmonary artery tumor, and pneumonectomy is performed for complete resection. However, it is difficult to see the inferior pulmonary vein and transect it safely via median sternotomy, so additional thoracotomy is often required and this is highly invasive. In the present case, we employed thoracoscopy (which we routinely use for VATS lobectomy) to transect the inferior pulmonary vein via median sternotomy without additional intercostal thoracotomy. This method has advantages for patients undergoing pneumonectomy via median sternotomy.

## Background

A right hilar tumor that completely occluded the right pulmonary artery was safely resected by pneumonectomy via median sternotomy with one-port thoracoscopic support. When surgery is performed via median sternotomy, additional intercostal thoracotomy is often needed to view the inferior pulmonary vein, resulting in a more invasive procedure. In this patient, a single thoracoscopic port allowed easy visualization of the inferior pulmonary vein and it was resected safely. The pathological diagnosis was pulmonary artery sarcoma, and the patient was discharged on postoperative day 16 after an uneventful course.

## Case presentation

**Fig. 1:**
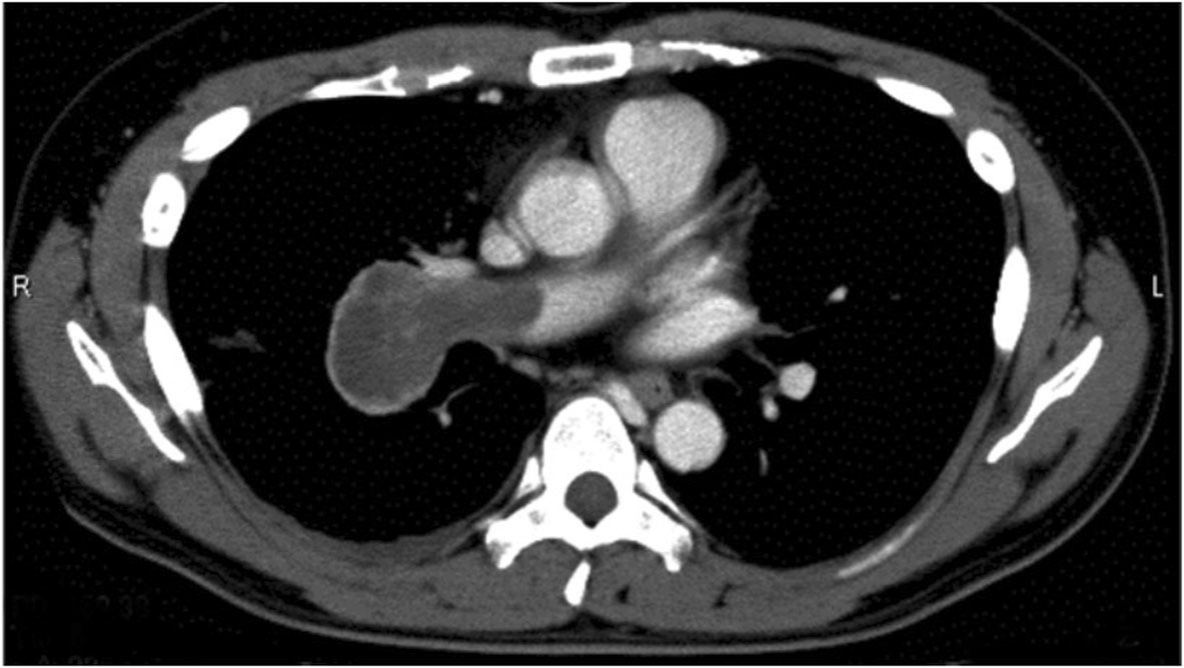
Contrast chest CT shows a right hilar mass that has invaded and occluded the right pulmonary artery

A 41-year-old man was referred to our hospital with the chief complaint of progressive dyspnea over 1 month. His vital signs were almost stable with oxygen saturation of 94% on room air. A chest X-ray film showed a right hilar mass lesion and enhanced computed tomography detected a mass that completely obstructed the right pulmonary artery (Fig. 1). There were also multiple nodules in both lungs, which were suspected to be metastatic lesions. Generally, we should obtain histological diagnosis of the pulmonary nodules to judge the operability by bronchofiberscopy or percutaneous needle biopsy. However, in this case, it would have been a risky procedure because of the respiratory insufficiency, and it might have been even critical if contralateral pneumothorax had been complicated after lung puncture by percutaneous needle biopsy. Regarding the main tumor, we consulted cardiologists to rule out pulmonary thrombosis and for histological diagnosis, but angiography was judged not to be indicated. Therefore, surgery was planned for both diagnosis and treatment.

The tumor was located in the right hilar region and was approached via median sternotomy. No pleural effusion or dissemination was detected in the ipsilateral thorax. At first, the right main pulmonary artery was encircled by approaching between superior vena cava and ascending aorta. However, main tumor was palpable and judged as impossible to transect it to ensure the surgical margin. Therefore, we tried to encircle the right main pulmonary artery on the left side of the ascending aorta. If it had been difficult to encircle it on the left side, we should have converted to the procedure under cardiopulmonary bypass. However, in this case, it was possible to encircle the vessel by blunt finger dissection and transected it with a surgical stapler. The superior pulmonary vein was also transected with a surgical stapler, but the inferior pulmonary vein could not be identified. Previously, we have added an intercostal incision to confirm the inferior pulmonary vein. However, we used a single thoracoscopic port (11.5 mm in diameter) placed at the 8th intercostal space on the mid-axillary line in the present patient. This allowed us to detect the inferior pulmonary vein easily, and we transected it safely with a surgical stapler, while avoiding additional intercostal thoracotomy and accomplishing tight pneumonectomy. The patient’s course was uneventful, and he was discharged on postoperative day 16. Nodules in the contralateral lung disappeared after surgery, suggesting that these were non-metastatic lesions.

**Fig. 2:**
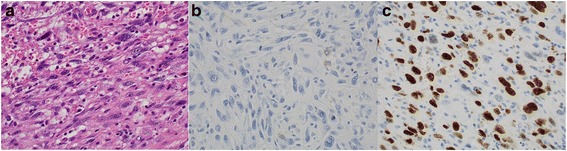
**a** Microscopy shows a hypercellular pattern of pleomorphic spindle cells and epithelioid cells with prominent nuclei and mitotic figures (hematoxylin and eosin stain). **b** Immunohistochemical staining displays focal positivity for alpha-SMA. **c** The Ki-67 proliferation index was 30%

Histopathological examination showed that the tumor extended into the lumen and also had spread beyond the pulmonary artery. Microscopy of hematoxylin and eosin stained sections revealed a hypercellular pattern of pleomorphic spindle cells and epithelioid cells, with prominent nuclei and mitotic figures. Immunohistochemical staining showed that the cells were focally positive for alpha-SMA and were negative for HHF35, CD34, c-kit, and osteopontin. The Ki-67 proliferative index was 30% (Fig. 2). Tumor was diagnosed as a pulmonary artery sarcoma, and residual tumor cells were detected at the surgical margin of the pulmonary artery. As adjuvant therapy, he received radiation (60 Gy) to the surgical stump and 4 cycles of chemotherapy (ifosfamide 2 mg/m^2^, epirubicin 45 mg/m^2^). The ipsilateral pulmonary nodules were suspected to be metastatic lesions but were found to be thrombosis, and there was no evidence of malignancy.

## Discussion

Primary pulmonary artery sarcoma is a rare disease, which was first reported in 1923 [1]. The prognosis is poor and a mean survival time of 1.5 months after diagnosis without surgical intervention has been reported. When resection is performed, the survival time varies from months to years and is determined by the presence of recurrence or metastasis. The location of the tumor and its hemodynamic effects also strongly influence the prognosis [2], and surgical treatment is often necessary.

In the present patient, two points of surgical interest should be discussed. The first is where the pulmonary artery should be resected. It is obvious that the pulmonary artery should be transected as proximally as possible, but the tumor extended to near the bifurcation of the main pulmonary trunk, so it was difficult to encircle the vessel with the stapler proximally enough. First, we encircled the pulmonary artery through the space between the superior vena cava and ascending aorta, but a safe surgical margin was not ensured due to proximal tumor extension. Therefore, we taped the vessel at this position and then encircled the pulmonary artery with the stapler for resection on the left side of the ascending aorta. Because residual tumor was detected at the margin, we should have resected the pulmonary artery more proximally using extracorporeal circulation, but metastases to the contralateral lung were suspected and we did not want to perform a more invasive procedure that would not be curative.

**Fig. 3:**
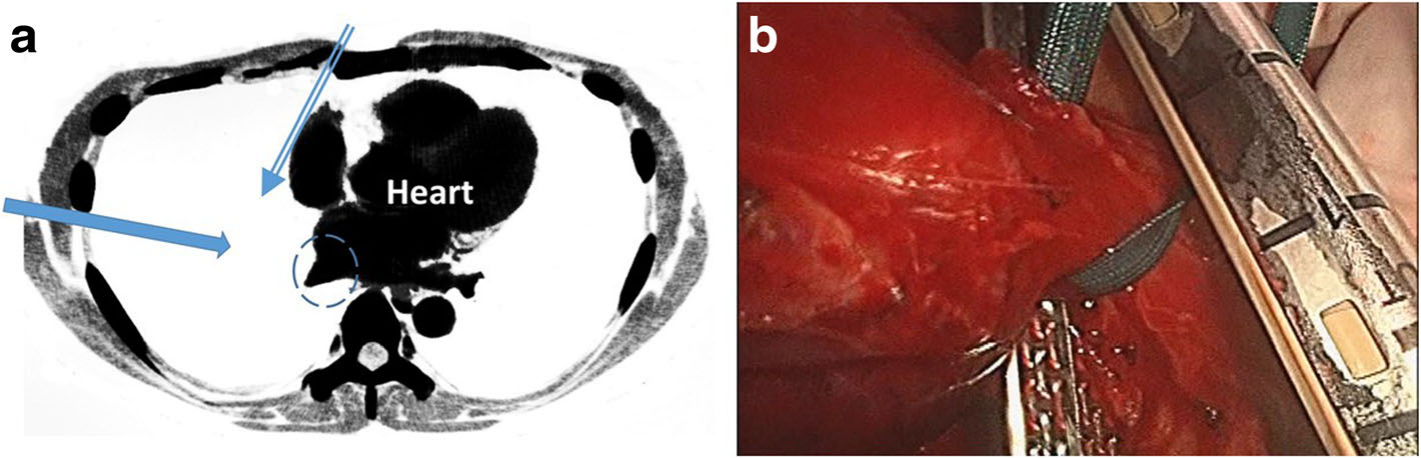
**a** Scheme showing visualization of the inferior pulmonary vein (*dotted circled line*) via median sternotomy or lateral thoracoscopy. It is difficult to directly visualize the inferior pulmonary vein via median sternotomy because it is blocked by the heart (*double line arrow*), but the vein can be easily identified by thoracoscopy (*single line arrow*). **b** Simple and safe transection of the inferior pulmonary vein with a mechanical stapler and thoracoscopic assistance

The second point is how to visualize the inferior pulmonary vein when resecting it. Generally, this vein is difficult to identify via median sternotomy because the heart blocks it from view. Sometimes we can see the inferior pulmonary vein by displacing the heart, but if this is not successful, intercostal thoracotomy is generally required to visualize the inferior pulmonary vein. Recently, we have performed many thoracoscopic lobectomy procedures. In this case, we used thoracoscopy to visualize the inferior pulmonary vein and resect it safely with a surgical stapler. Thus, we avoided additional intercostal thoracotomy and the surgical procedure was less invasive for the patient (Fig. 3).

Pulmonary artery sarcoma is rare and the diagnosis is usually made postoperatively or at autopsy [3]. Recently, intravenous catheter suction biopsy has been attempted for diagnosis of this tumor at some centers [4] and it has been reported that atypical cells could be detected, but pathological diagnosis was not possible. We also consulted cardiologists, but they decided not to perform intravenous biopsy and recommended surgery. Since the patient has multiple pulmonary nodules in both lungs, which were suspected to be metastases, it was difficult to decide on surgical resection because there seemed to be no chance of radical cure. However, a pathological diagnosis was necessary to select treatment, so we decided to perform surgical resection of the tumor and right pneumonectomy. In patients with this tumor, the main pulmonary artery has been replaced by a homograft and the pulmonary valve may also be replaced [5]. In our patient, residual tumor was detected at the resection margin of the pulmonary artery, indicating that more extensive pulmonary artery resection with extracorporeal cardiopulmonary support would have been preferable. However, we decided not to perform more invasive resection because the multiple bilateral pulmonary nodules were suspected to be metastases.

## Conclusions

Pulmonary artery sarcoma is a rare tumor, and it is difficult to obtain a definite diagnosis. Because the outcome is very poor without radical resection, this disease generally requires surgical treatment. Even with radical resection, the prognosis is generally unsatisfactory, so we have to make efforts to select less invasive treatment modalities for these patients.
